# Exploring the impact of 3,3’-diindolylmethane on the urinary estrogen profile of premenopausal women

**DOI:** 10.1186/s12906-024-04708-7

**Published:** 2024-11-22

**Authors:** Mark Newman, Jaclyn Smeaton

**Affiliations:** Precision Analytical, Inc., McMinnville, USA

**Keywords:** Estrogen metabolites, Estradiol, Dietary supplement, DIM, I3C, Dried urine

## Abstract

**Background:**

3,3’-diindolylmethane (DIM) is a phytonutrient derived from cruciferous vegetables that is an often-used supplement in the complementary and alternative medicine space. The most common goal for providers when recommending DIM to their patients is to alter estrogen metabolism, yet research into DIM’s effect on the estrogen profile is lacking in the published literature. The objective of this study was to comprehensively evaluate DIM’s effect on the urinary estrogen profile.

**Methods:**

In this retrospective cohort study, we analyzed data from a clinical laboratory, including urinary estrogen and estrogen metabolite concentrations. Analyte concentrations were determined from dried urine samples using a gas chromatography-tandem mass spectrometry assay. Individuals were separated into two groups, either reporting taking DIM (*N* = 909) or reporting not taking DIM (*N* = 18,385). Comparisons between individuals in these two groups were made using the Wilcoxon rank sum test. Additionally, we were also able to explore a subset of women who had laboratory results in the database before and after initiating DIM treatment (*N* = 53). In this subset, differences were assessed with Wilcoxon signed rank tests.

**Results:**

In the larger group that was separated into women reporting either DIM use or no use, significant differences were observed in the concentrations of almost every urinary estrogen and estrogen metabolite (with the only exception being 2-methoxyestrone) in the urinary estrogen profiles of those taking DIM compared to those not taking DIM (all P values < 0.001). In the smaller subset of individuals with results before and after initiating DIM use, differences were only seen in 4 of the urinary estrogens and estrogen metabolites (*P* < 0.001 for estrone, estradiol, estriol, and 16-hydroxyestrone). Differences in total estrogens were significant in both the larger group and the smaller subset (both with *P* < 0.001). Additionally, observed differences in the ratios of metabolites followed a similar trend with more significant differences observed in the larger group. Notably, the 2-hydroxyestrone:16-hydroxyestrone ratio increased significantly in both the larger group and the smaller subset with results before and after DIM use.

**Conclusions:**

The results of this study provide the most comprehensive evaluation to date of DIM’s effect on the urinary estrogen profile. Additionally, the results demonstrate that the dried urine collection and accompanying assay used capture changes that are similar in direction, but not necessarily magnitude, to previous reports in the literature. Considered together, these two things highlight the clinical validity and utility of this approach to the evaluation of DIM supplementation and suggest the need for additional studies using this approach to fully understand the potential clinical utility of DIM.

## Introduction

As part of the burgeoning interest in integrative and functional medicine approaches to women’s health, dietary supplements such as indole-3-carbinol (I3C) and 3,3’-diindolylmethane (DIM) have gained attention for their potential impact on estrogen metabolism. I3C and DIM are derived from cruciferous vegetables including broccoli, Brussels sprouts, bok choy, cabbage, cauliflower, collards, and kale [1]. Once ingested, I3C is rapidly converted to multiple oligomers of which DIM is the predominant active agent, and the molecule responsible for the majority of the observed effects [1–3]. However, although considerable experimental and theoretical effort has been aimed at understanding the mechanism of action (MOA) and effects of DIM in other contexts [4–10], little focus has been directed at understanding its MOA and effects in applications common to functional and integrative medicine.

The MOA of DIM is complex and not completely understood. When recommending DIM supplements to patients, the goal of integrative and functional medicine providers is usually to alter estrogen metabolism or lower total estrogen levels. In this setting, the most relevant component of DIM’s MOA is the modulation of estrogen metabolism via the induction and inhibition of cytochrome P450 (CYP) enzymes. In previous studies, DIM was shown to be an inducer of CYP1A1, CYP1A2, and, to a lesser extent, CYP3A4 [11, 12]. Importantly, DIM’s MOA has many other proposed components that aren’t directly relevant to the current study.

In order to understand the potential impact of DIM on estrogen metabolism, it is necessary to understand the role of CYP enzymes in estrogen metabolism. In phase 1 of estrogen metabolism estrone (E1) and estradiol (E2) undergo hydroxylation reactions which are mediated by the same CYP enzymes that DIM induces, along with CYP1B1, and to a lesser extent CYP3A5 and CYP3A7 [13]. CYP1A1 and CYP1A2 catalyze the hydroxylation of E1 and E2, leading to the formation of 2-hydroxyestrone (2-OHE1) and 2-hydroxyestradiol (2-OHE2) which are considered to be less proliferative compared to other phase 1 metabolites [12, 14]. In contrast, CYP1B1 catalyzes the 4-hydroxylation of E1 and E2 producing 4-hydroxyestrone (4-OHE1) and 4-hydroxyestradiol (4-OHE2), which are considered to be more proliferative metabolites due to the reactive quinones they can yield via redox cycling [12]. The 4-hydroxylation reaction is also catalyzed, to a lesser extent, by CYP3A4 and CYP3A5 [13].

In addition to there being little investigation of the MOA of DIM as it relates to integrative and functional medicine indications, there is similarly little research into understanding the effects of DIM on the entire estrogen profile. The studies that have been published generally only look at one component of the estrogen profile that is of interest, such as the 2-OHE1/16-OHE1 ratio [15, 16]. This fact has presented functional and integrative medicine practitioners with a dilemma. Despite compelling anecdotal evidence of the effectiveness of DIM in integrative and functional medicine applications, there are no published studies that provide definitive evidence of the expected changes in the estrogen profile. Compounding this issue is the fact that the mechanism of action of DIM is not completely understood.

The dearth of direct evidence in the published literature urged us to explore potential mechanisms that may be illuminated in the urinary estrogen profile. Previous studies have validated the collection and assay method used in the current study for exploring estrogen metabolism in other contexts [17–21]. The primary aim of this study was to use this validated method to explore and present a detailed analysis of DIM’s effects on estrogen metabolism in humans by evaluating the complete urinary estrogen profile of premenopausal women in the luteal phase of their cycle. The complete urinary estrogen profile we sought to explore includes parent estrogens, estrogen metabolites, and relevant ratios.

## Materials and methods

### Data source

In this retrospective data mining study registered as Precision Analytical Retrospective Data Correlation (Clinical Trials ID, NCT04305093), data were available for 144,561 laboratory accessions from 129,883 individuals between January 1, 2016 and December 9, 2019. The study was approved by the National University of Natural Medicine Institutional Review Board, and since all data analyzed in this data mining study were deidentified, the institutional review board determined that written informed consent could be waived. Deidentified data were extracted from the database and included the following variables: age, sex, menstrual status (regular, irregular, none), last menstrual period, body mass index (BMI) calculated from self-reported height and weight, use of DIM, comorbidities, and urinary measures of estrone, estradiol, estriol, 𝛼 and 𝛽-pregnanediol, 2-hydroxyestrone, 2-hydroxyestradiol, 4-hydroxyestrone, 4-hydroxyestradiol, 16-hydroxyestrone, and 2-methoxyestrone. Data on race/ethnicity were not available. Inclusion criteria for this study included being a premenopausal woman reporting regular menses and having collected samples in the luteal phase of the menstrual cycle. Exclusion criteria included the following: a BMI less than 16 or greater than 60 kg/m^2^; pregnancy; any reported kidney disease; reported adrenal insufficiency or excess; suspected or diagnosed PCOS; missing data for urinary estrogens or estrogen metabolites (with the exception of 4-hydroxyestradiol and 2-methoxyestradiol); use of any estrogen supplementation (oral, sublingual pelleted, injected, or other transdermal formulation); use of 𝛽-human chorionic gonadotropin; use of anastrazole; use of tamoxifen; evidence of overly dilute urine (any spot urine creatinine < 0.1 mg/ml).

### Sample collection and laboratory methods

The sample collection and assay methods used to produce the data analyzed in this study, including assay performance characteristics, have been described previously [17, 18]. Briefly, participants collected a total of four urine samples on filter paper over 24 h. These samples were allowed to dry and then shipped to the laboratory for analysis. Once samples were received in the laboratory, urine was extracted from the filter paper and the four samples were combined into a single representative sample. This combination was done by using creatinine concentrations from each individual sample to determine the volume of sample that should be added to the combined sample to best represent measured concentrations over a 24-hour period. The combined sample was then analyzed via gas chromatography-tandem mass spectrometry on an Agilent 7890/7000B (Agilent Technologies, Santa Clara, CA). Estrogen and estrogen metabolite concentrations were normalized to creatinine to account for variation in both urine concentration and filter paper saturation. Urinary sample collection was the same for all participants in any group (women reporting DIM use or no use, and for women before and after initiating DIM).

### Statistical methods

All values are presented as medians and interquartile ranges as they were not normally distributed. In the larger group, we compared characteristics of premenopausal women taking DIM and premenopausal women not taking DIM. These comparisons were done using the Wilcoxon rank-sum test. In the smaller subset of individuals who had laboratory results available both before and after initiating DIM, comparisons were made with the Wilcoxon signed rank test. Because this was a data mining study using a convenience sample, no sample size or power calculation was performed a priori, and all comparisons should be considered exploratory and hypothesis generating in nature. All analyses were conducted using R version 4.3.1 (R Version for Statistical Computing, Vienna, Austria).

## Results

### Comparison of women not taking DIM to women taking DIM

After applying inclusion and exclusion criteria, there were 18,385 women who did not report taking DIM and 909 women who reported taking DIM. Patient characteristics and urinary estrogen and estrogen metabolite concentrations for these two groups of women are reported and compared in Table 1. The urinary estrogen metabolite ratios for these two groups are reported and compared in Table 2. Significant differences were observed in both age and BMI along with all urinary estrogen metabolites with the exception of 2-methoxyestrone. Urinary E1, E2, and E3 concentrations were all lower in the group taking DIM, as were the concentrations of 16-hydroxyestrone and total urinary estrogens. Concentrations of 2-hydroxyestrone, 4-hydroxyestrone, 2 hydroxyestradiol, and 4-hydroxyestradiol were all higher in the women taking DIM. These differences in metabolite concentrations translated to significant differences in all of the metabolite ratios that were calculated.

**Table 1:** Patient characteristics and urinary estrogen concentrations

Characteristic	No DIM, *N* = 18,385^1^	DIM, *N* = 909^1^	*p*-value^2^
Age at Collection	38 (33, 43)	40 (35, 44)	< 0.001
BMI	23.8 (21.3, 26.6)	23.4 (21.0, 25.8)	0.003
Urinary Estrone (ng/mg-Cr)	18.00 (12.70, 24.90)	14.70 (10.30, 20.70)	< 0.001
Urinary Estradiol (ng/mg-Cr)	3.10 (2.22, 4.34)	2.62 (1.89, 3.86)	< 0.001
Urinary Estriol (ng/mg-Cr)	9.60 (5.90, 15.10)	5.20 (3.00, 9.50)	< 0.001
Urinary 2-hydroxyestrone (ng/mg-Cr)	8.24 (5.40, 11.50)	11.55 (7.90, 15.81)	< 0.001
Urinary 4-hydroxyestrone (ng/mg-Cr)	1.09 (0.75, 1.50)	1.37 (0.97, 2.00)	< 0.001
Urinary 16-hydroxyestrone (ng/mg-Cr)	1.21 (0.73, 1.99)	0.82 (0.45, 1.44)	< 0.001
Urinary 2-methoxyestrone (ng/mg-Cr)	4.20 (2.79, 5.91)	4.05 (2.70, 5.97)	0.7
Urinary 2-hydroxyestradiol (ng/mg-Cr)	0.66 (0.36, 1.09)	0.99 (0.55, 1.58)	< 0.001
Urinary 4-hydroxyestradiol (ng/mg-Cr)	0.30 (0.20, 0.40)	0.40 (0.20, 0.50)	< 0.001
Missing Values	5,560	260	
Total Urinary Estrogens (ng/mg-Cr)	48.50 (36.50, 63.30)	44.20 (33.00, 58.10)	< 0.001

^1^Median (IQR)

^2^Wilcoxon rank sum test

**Table 2:** Patient characteristics and urinary estrogen metabolite ratios

Characteristic	No DIM, *N* = 18,385^1^	DIM, *N* = 909^1^	*p*-value^2^
Age at Collection	38 (33, 43)	40 (35, 44)	< 0.001
BMI	23.8 (21.3, 26.6)	23.4 (21.0, 25.8)	0.003
2OHE1/16OHE1 Ratio	6.89 (3.72, 12.10)	15.36 (7.49, 25.65)	< 0.001
Missing Values	1	0	
2OHE1/E1 Ratio	0.44 (0.31, 0.61)	0.79 (0.54, 1.10)	< 0.001
Missing Values	1	0	
2OHE1/E2 Ratio	2.56 (1.69, 3.63)	4.30 (2.87, 5.99)	< 0.001
2OHE1/4OHE1 Ratio	7.27 (5.36, 9.46)	8.15 (6.00, 10.43)	< 0.001
Missing Values	2	0	
2-methoxyE1/2OHE1 Ratio	0.51 (0.40, 0.66)	0.35 (0.27, 0.46)	< 0.001
2OHE2/2OHE1 Ratio	0.08 (0.06, 0.11)	0.08 (0.06, 0.12)	0.004

^1^Median (IQR)

^2^Wilcoxon rank sum test

### Before and after initiating DIM

**Fig. 1:**
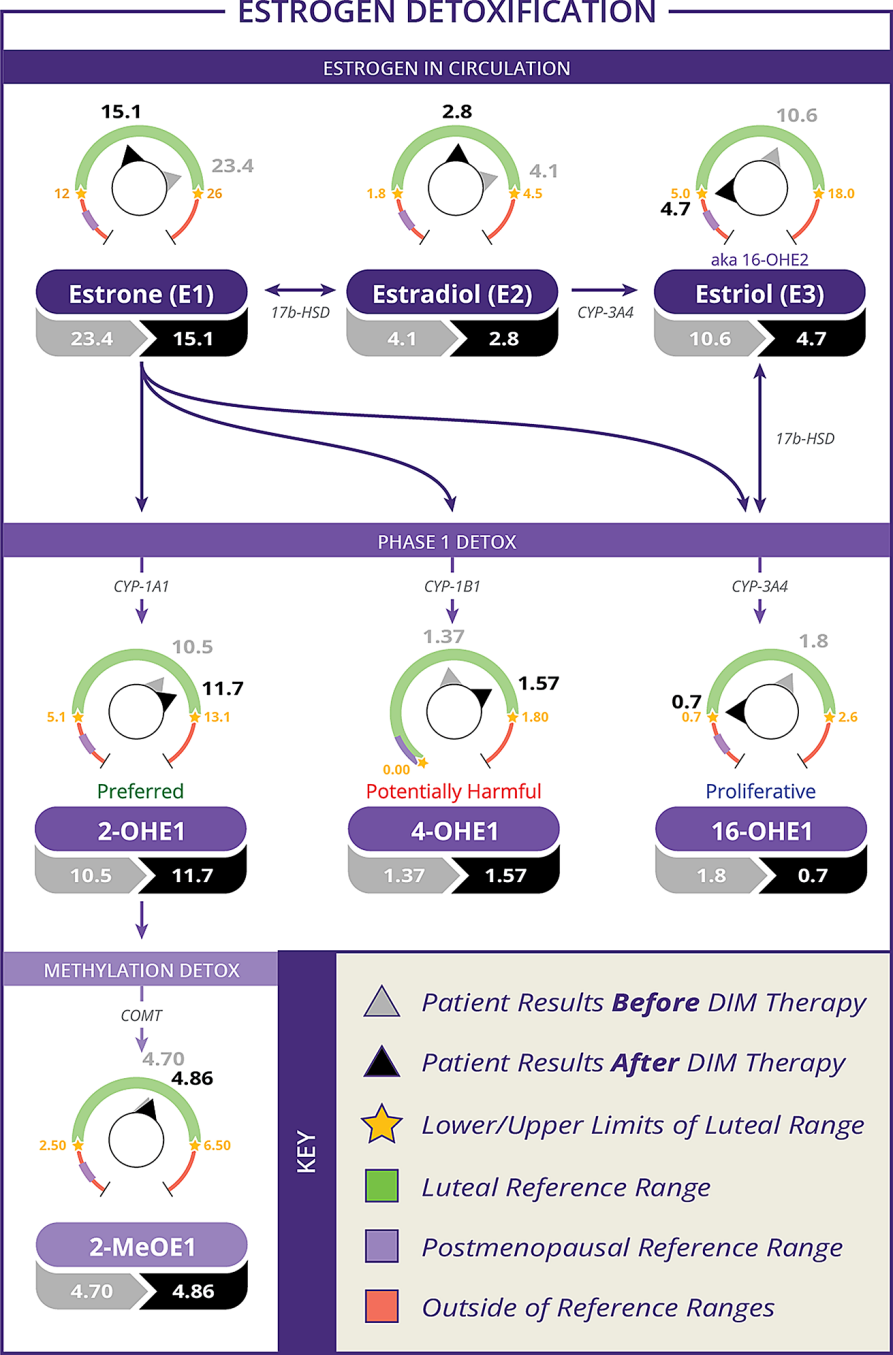
Urinary estrogen metabolite before and after values. Shown are values before and after DIM therapy for some of the estrogen metabolites and ratios in the context of the estrogen metabolic pathway

**Fig. 2:**
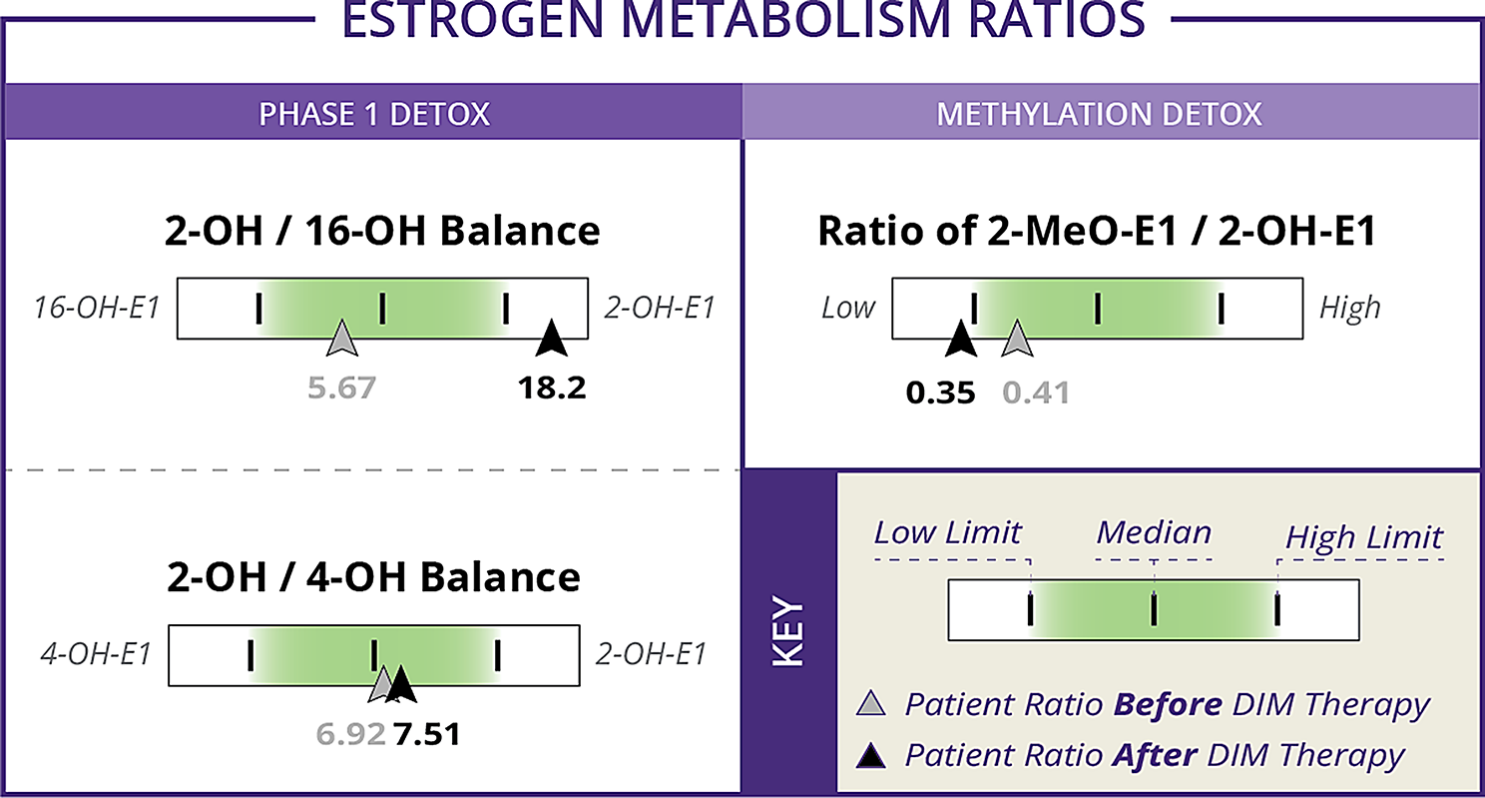
Urinary estrogen metabolite ratio before and after values. Shown are values before and after DIM therapy for estrogen metabolite ratios during phase 1 and methylation detox

**Fig. 3:**
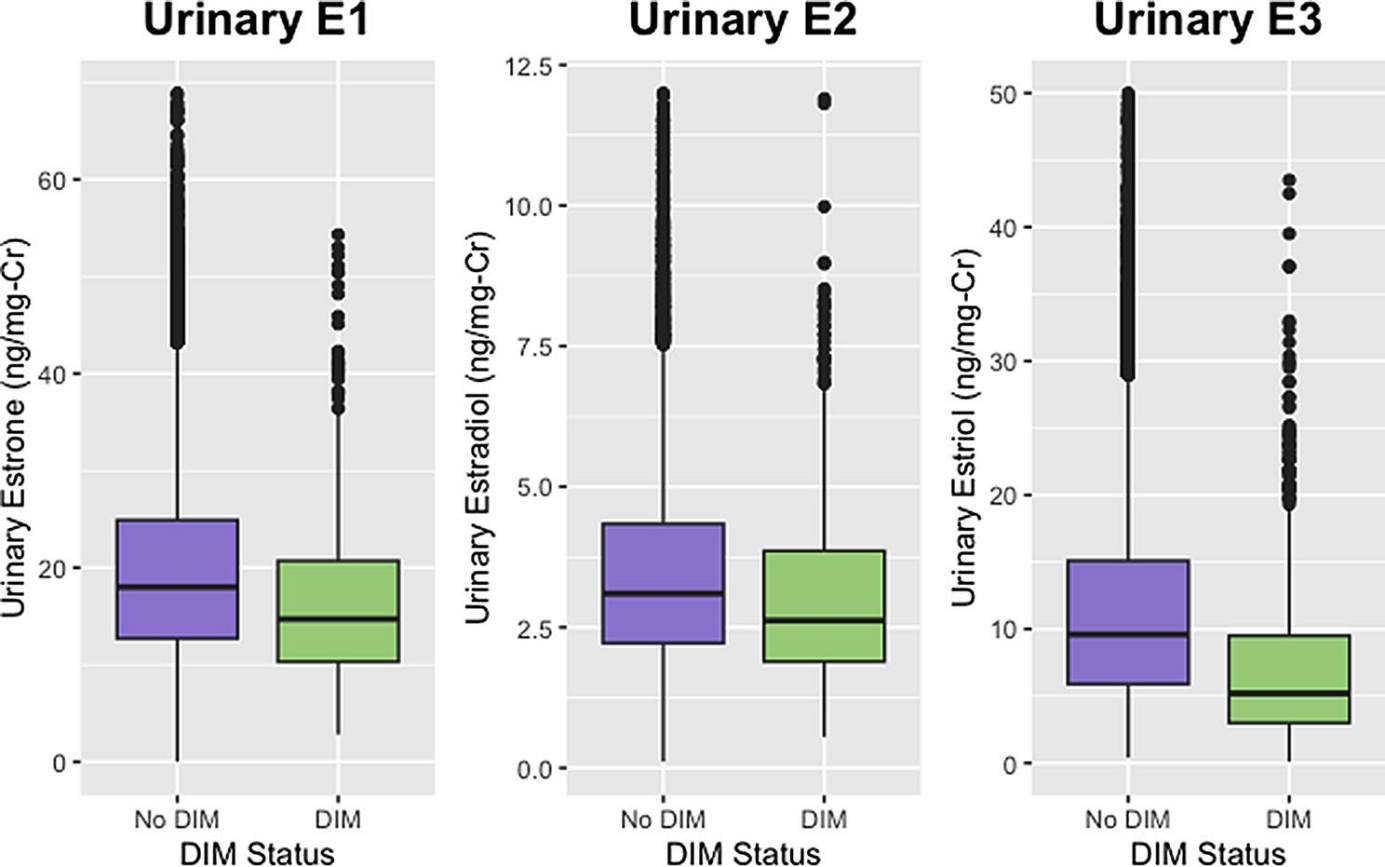
Comparison of distributions of E1, E2, and E3. Shown are urinary estrone (E1), estradiol (E2), and estriol (E3) in women reporting use of DIM or no use of DIM

**Fig. 4:**
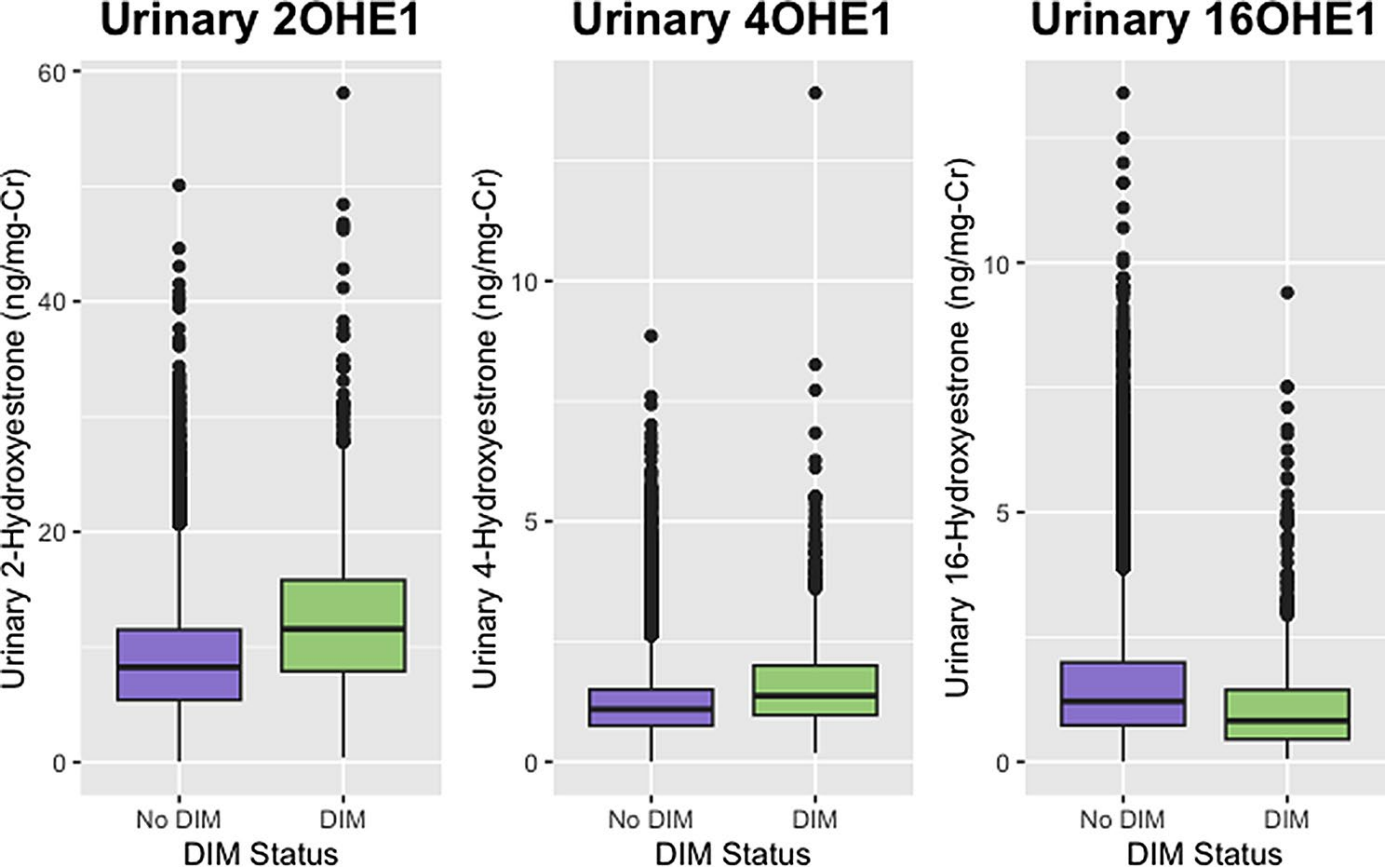
Comparison of distributions of selected metabolites. Shown are urinary 20HE1, 4OHE1, and 16OHE1 in women reporting use of DIM or no use of DIM

**Fig. 5:**
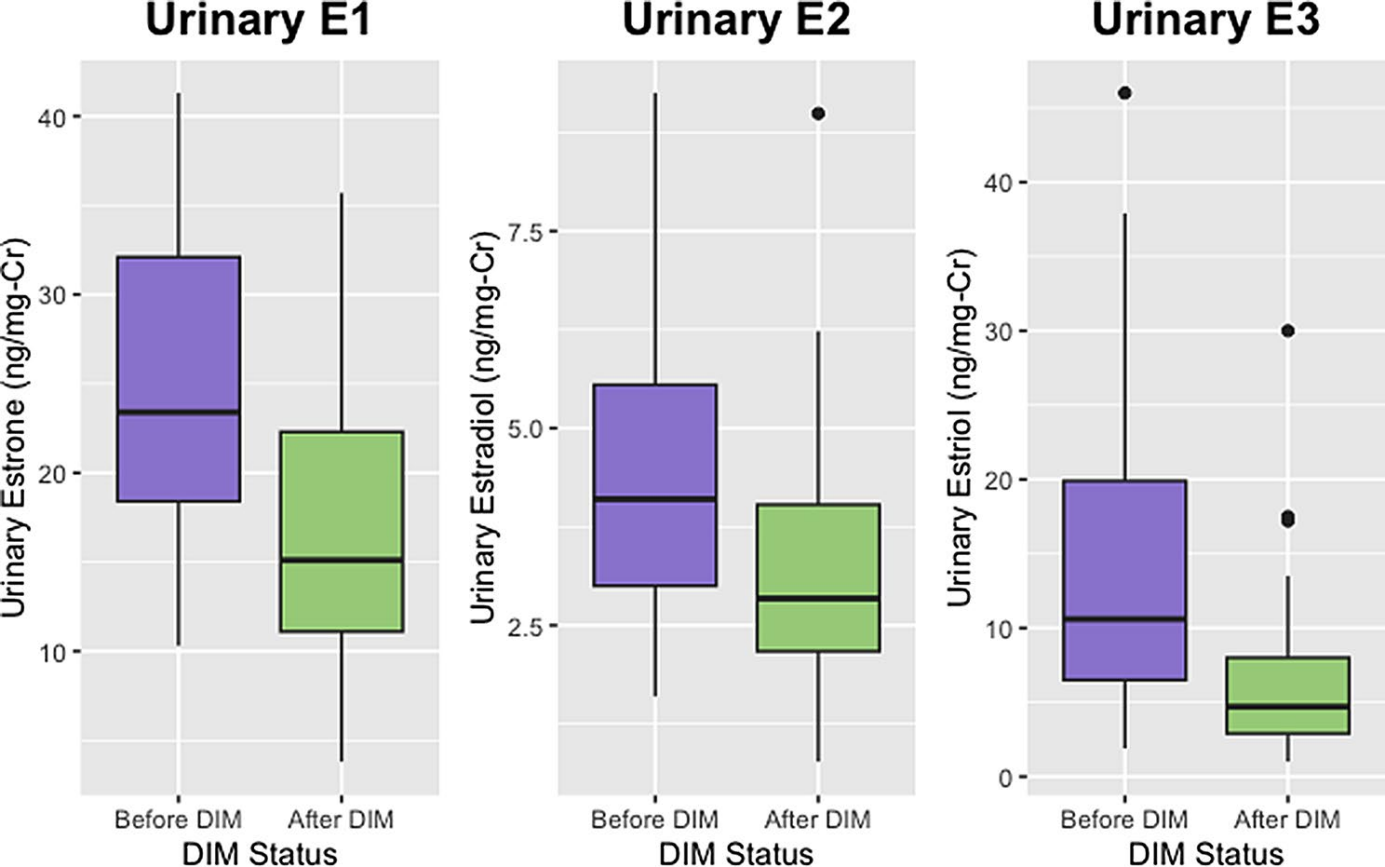
Comparison of distributions of E1, E2, and E3 (before and after DIM). Shown are urinary estrone (E1), estradiol (E2), and estriol (E3) in a subset of women before and after initiating DIM therapy

**Fig. 6:**
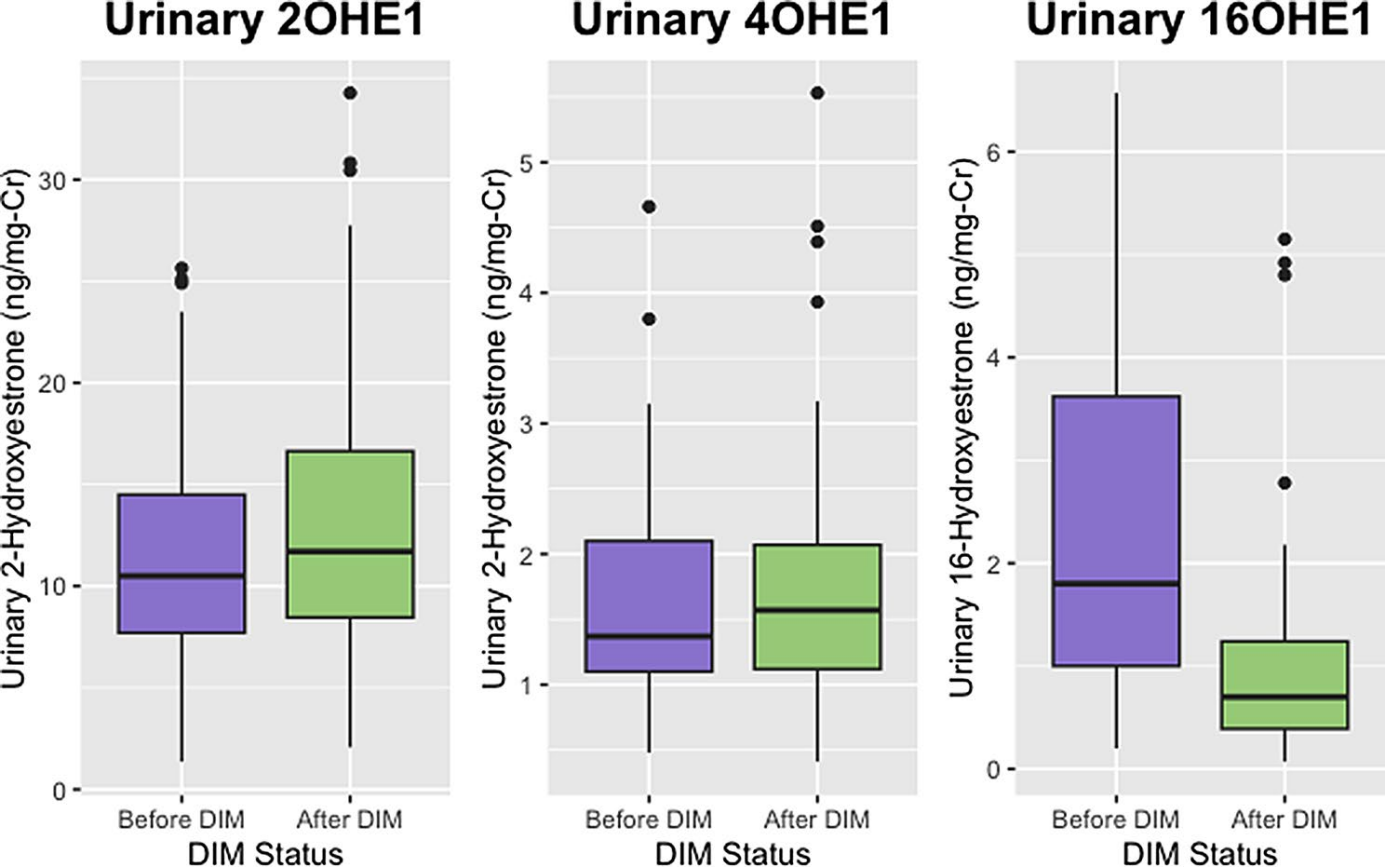
Comparison of distributions of selected metabolites (before and after DIM). Shown are urinary 20HE1, 4OHE1, and 16OHE1 in a subset of women before and after initiating DIM therapy

There was a total of 53 women who had laboratory results available both before and after starting DIM. Age, BMI, and urinary estrogen and estrogen metabolite concentrations for these women are reported and compared in Table 3. Urinary estrogen metabolite ratios for these women before and after initiating DIM are compared in Table 4. The before and after values for some of the metabolites and some of the ratios can be seen in the context of the estrogen metabolic pathway in Figs. 1 and 2. With the exception of 2-methoxyestrone and 2OHE2, all parent estrogens and metabolites changed in the same direction as in the larger group (Figs. 3, 4, 5 and 6). However, only the changes in E1, E2, E3, and 16-OHE1 reached the threshold for significance. All of the ratios changed in the same direction as in the larger group; however, the only ratio changes that reached significance were the 2-OHE1/16-OHE1, 2-OHE1/E1, 2-OHE1/E2, and the 2-methoxyE1/2-OHE1 ratios. The percent changes for E2, 2-OHE1, 4-OHE1, 16-OHE1, and the 2/16 ratio are presented in Table 5.

**Table 3:** Before and after estrogen & estrogen metabolite concentrations

Variable	Before DIM^1^	After DIM^1^	*p*-value^2^
Age at Collection	39 (32, 42)	40 (34, 44)	0.2
BMI	24.1 (21.3, 26.6)	23.4 (21.3, 25.7)	0.8
Urinary a-pregnanediol (ng/mg-Cr)	417 (284, 843)	427 (308, 681)	0.6
Urinary b-pregnanediol (ng/mg-Cr)	1,350 (1,144, 1,657)	1,263 (973, 1,964)	0.7
Urinary Estrone (ng/mg-Cr)	23.40 (18.40, 32.10)	15.10 (11.10, 22.30)	< 0.001
Urinary Estradiol (ng/mg-Cr)	4.10 (3.00, 5.55)	2.84 (2.17, 4.03)	< 0.001
Urinary Estriol (ng/mg-Cr)	10.60 (6.50, 19.90)	4.70 (2.90, 8.00)	< 0.001
Urinary 2-hydroxyestrone (ng/mg-Cr)	10.50 (7.70, 14.50)	11.70 (8.46, 16.64)	0.4
Urinary 4-hydroxyestrone (ng/mg-Cr)	1.37 (1.10, 2.10)	1.57 (1.12, 2.07)	0.5
Urinary 16-hydroxyestrone (ng/mg-Cr)	1.80 (1.00, 3.62)	0.70 (0.39, 1.24)	< 0.001
Urinary 2-methoxyestrone (ng/mg-Cr)	4.70 (3.70, 6.20)	4.86 (3.20, 6.12)	0.8
Urinary 2-hydroxyestradiol (ng/mg-Cr)	1.10 (0.51, 1.86)	1.01 (0.58, 1.68)	0.8
Urinary 4-hydroxyestradiol (ng/mg-Cr)	0.35 (0.30, 0.50)	0.40 (0.30, 0.60)	0.6
Missing Values	37	5	
Urinary 2-methoxyestradiol (ng/mg-Cr)	0.55 (0.48, 0.83)	0.50 (0.30, 0.73)	0.4
Missing Values	37	5	
Total Urinary Estrogens (ng/mg-Cr)	64.0 (50.3, 77.0)	47.2 (35.3, 60.7)	< 0.001

^1^Median (IQR)

^2^Wilcoxon rank sum test

**Table 4:** Before and after estrogen metabolite ratio values

Variable	Before DIM^1^	After DIM^1^	*p*-value^2^
Age at Collection	39 (32, 42)	40 (34, 44)	0.2
BMI	24.1 (21.3, 26.6)	23.4 (21.3, 25.7)	0.8
2OHE1/16OHE1 Ratio	5.67 (2.79, 13.66)	18.20 (9.64, 31.58)	< 0.001
2OHE1/E1 Ratio	0.45 (0.30, 0.61)	0.88 (0.56, 1.14)	< 0.001
2OHE1/E2 Ratio	2.40 (1.59, 3.81)	4.27 (3.05, 5.80)	< 0.001
2OHE1/4OHE1 Ratio	6.92 (5.41, 9.33)	7.51 (6.21, 9.69)	0.3
2-methoxyE1/2OHE1 Ratio	0.41 (0.32, 0.57)	0.35 (0.27, 0.51)	0.027
2OHE2/2OHE1 Ratio	0.09 (0.07, 0.12)	0.08 (0.07, 0.11)	0.6

^1^Median (IQR)

^2^Wilcoxon rank sum test

**Table 5:** Percent change in key metabolites and the 2/16 ratio (in women with results before and after initiating DIM)

Variable	Percent Change
Estradiol	-23.13%
2-Hydroxyestrone	19.24%
4-Hydroxyestrone	8.95%
16-Hydroxyestrone	-55.56%
2OHE1/16OHE1 Ratio	188.31%

## Discussion

In this study, we have examined retrospective data in an attempt to more completely understand the effect of DIM on estrogen metabolism in premenopausal women. We found significant differences in almost all estrogens, estrogen metabolites, and estrogen metabolite ratios when we compared the larger groups of women who were either taking DIM or not taking DIM. In the smaller group of women who had laboratory results available before and after starting DIM, although we saw differences in the same directions as in the larger group, fewer of these differences reached significance.

This study has several limitations. The data used in this analysis is from a convenience sample and thus there is limited external generalizability to populations that are different from the population studied. However, given the large sample size, valuable insights can be garnered to inform the design of future, more targeted studies. We also had no access to data on participants’ diet which could have a meaningful impact if a participant’s diet was particularly high in cruciferous vegetables (or particularly low). In addition to not having access to data on diet, although we did collect some data regarding prescription medication and supplement use, we were unable to collect an exhaustive list of concurrent prescription medications and over the counter supplements. In this patient population there is sometimes concurrent use of other estrogen-lowering or estrogen metabolism altering agents such as calcium-d-glucarate [22]. These agents may have also contributed to some of the lower estrogen and estrogen metabolites concentrations seen, but these agents would be less likely to affect the ratios of estrogen metabolites. We did not collect data regarding dose or formulation of DIM/I3C, frequency of administration, or adherence. Although this is certainly a limitation, it also reflects the real-world nature of the data and represents what is likely seen in actual clinical practice. There are DIM products which claim higher bioavailability [23], so it is possible that the use of different products may have significantly different impacts on the urinary estrogen profile. The results of this study highlight that this collection method and assay would be a useful tool to investigate potential differing impacts between DIM products. Because of differing bioavailability and half-life, some products may recommend more or less frequent dosing, however dosing is usually once or twice daily. Finally, we do not have data on the rationale employed for initiating DIM. The reasons for using DIM vary widely in the integrative and functional medicine space, and capturing the potential “indications” for DIM use concisely is beyond the scope of this manuscript. Notwithstanding these limitations, this study provides both a comparative evaluation of premenopausal women taking vs. not taking DIM and a repeated measures evaluation of a subset of women using DIM. These evaluations shed light on the impact of DIM on the entire urinary estrogen profile which, to the best of our knowledge, has not previously been explored this comprehensively.

Many of the previous studies that evaluated the effects of DIM focused on changes in 2-OHE1, 16-OHE1, and the 2/16 ratio [6, 15, 16, 24]. In addition to the focus on the 2-OHE1 and 16-OHE1, Dalessandri and colleagues also reported urinary E1, E2, and E3 concentrations before and after DIM use, along with urinary 6𝛽-hydroxycortisol (6𝛽-OHC), cortisol, and the 6𝛽-OHC/cortisol ratio, which has shown to be an indication of CYP3A4 enzyme activity [25]. As discussed earlier, regarding DIM, the activity of CYP3A4 influences production of 16-OHE1 and 4-OHE2, the non-favored estrogen metabolites. One of the only studies in the literature that is similar to the present study is one done by Green and colleagues which used the same assay as the present study [16]. However, participants in that study were given a dietary supplement that contained I3C and at least 8 additional ingredients. Moreover, the women participating in that study may not have been adequately fluid restricted and samples were collected during the day. The combination of these 2 factors may have led to low creatinine values to which the estrogen metabolite concentrations are indexed. Furthermore, the overall estrogen and estrogen metabolite concentrations for the study participants were low compared to established reference ranges. The primary aim of that study was simply to determine if the nutraceutical product used, Estrosense, increased the 2/16 ratio.

Results in this study are similar in direction to the four previously mentioned studies conducted by Rajoria and colleagues, Thomson and colleagues, Godinez-Martinez and colleagues, and Green and colleagues. Here, just as in all of those studies, 2-OHE1 increased and 16-OHE1 decreased with DIM treatment, which translated to an increase in the 2/16 ratio. One interesting observation concerns the change in 4-OHE1. In the present study, although 4-OHE1 increased in the before and after group, the change did not reach significance. Similar results were seen in the study by Thomson and colleagues. They reported an initial small median decrease at 6 months, followed by an increase at 12 months; however median change from baseline to 12 months was 0.0, and the P-value comparing DIM treated participants with the placebo arm was not significant (*P* = 0.534) indicating no difference in effect on 4-OHE1 between DIM and placebo. This echoes the conclusion that we have drawn from our data. This conclusion may be surprising to some integrative and functional medicine providers who expect DIM to reduce 4-OHE1. The currently available evidence does not support DIM use for this purpose. The 2OHE1/4OHE1 ratio did increase in both groups, although this change did not reach the threshold for significance in the before and after group.

Interestingly, in the study conducted by Green and colleagues, the only outcome measures with changes from baseline that reached statistical significance were 2-OHE1 (*P* = 0.01) and the 2/16 ratio (*P* < 0.001). This indicates that the change in the primary outcome was driven by the observed increased in 2-OHE1 which was only 0.43 ng/mg-Cr. In the present study we saw a somewhat opposite result in that even though the median 2-OHE1 concentration increased by a larger margin (1.20 ng/mg-Cr), the difference from baseline was not significant (*P* = 0.4). However, the difference in 16-OHE1 was significant (*P* < 0.001). This suggests that although the outcome measure of increase in the 2/16 ratio was the same in both studies, the underlying change in estrogen metabolism driving the increase in the ratio was different in these two studies. This has important implications for clinical decision making depending on the goals of the provider.

Future randomized, placebo-controlled studies with multiple DIM products are needed to fully understand the potential clinical utility of DIM supplementation. The tools presented here for assessing the effects of DIM seem to be the most comprehensive approach to gaining actionable clarity.

## Conclusion

Although a complete understanding of the mechanism of action of DIM continues to elude researchers, this and previous studies serve to illuminate some of the expected effects in premenopausal women. The results of this study demonstrate that the dried urinary sampling method and corresponding assay used to measure estrogen metabolites is a useful approach to monitoring the effects of DIM therapy in real world clinical practice. To conclude, we propose that the changes seen here in parent estrogen concentrations, estrogen metabolite concentrations, and estrogen metabolite ratios represent a key component to understanding the complex and somewhat convoluted mechanism of action of DIM. The specific changes that we have either confirmed or uncovered provide a molecular explanation for some of the observed effects of DIM. The results of this study, taken together with the theoretical and observed partial mechanism of action of DIM, provide moderate evidence supporting the potential clinical utility of DIM and strong evidence supporting the definite clinical utility of urinary estrogen and estrogen metabolite monitoring in the setting of DIM supplementation. Additional work is needed to completely reveal how the complex mechanism of action of DIM is reflected in the complex estrogen metabolism pathway. Disentangling the complexity of estrogen metabolism remains a formidable challenge, which may well demand the use of novel methods. We posit that the use of urinary estrogen and estrogen metabolite concentration changes to explore theoretical and actual consequences of altered estrogen metabolism will prove to be a drama of refound opportunity in the coming years.

## Data Availability

The datasets used and/or analyzed during the current study are available from the corresponding author on reasonable request.
